# Supplementary material for: The contributions from the progenitor genomes of the mesopolyploid Brassiceae are evolutionarily distinct but functionally compatible
Source: Genome Res. 2021 May;31(5):799–810. doi: 10.1101/gr.270033.120 (PMC8092008; doi:10.1101/gr.270033.120)
Supplement: Supplemental Material [file supp_31_5_799__index.html]

The contributions from the progenitor genomes of the mesopolyploid Brassiceae are evolutionarily distinct but functionally compatible — Supplemental Material 

# The contributions from the progenitor genomes of the mesopolyploid Brassiceae are evolutionarily distinct but functionally compatible

## Supplemental Material

- Supplemental\_Data.zip
- Supplemental\_Code.zip
- Supplemental\_Information.pdf
