# Supplementary material for: The contributions from the progenitor genomes of the mesopolyploid Brassiceae are evolutionarily distinct but functionally compatible
Source: Genome Res. 2021 May;31(5):799–810. doi: 10.1101/gr.270033.120 (PMC8092008; doi:10.1101/gr.270033.120)
Supplement: Supplemental Material [file supp_gr.270033.120_Supplemental_Information.pdf]

Supplemental Information

The contributions from the progenitor genomes of the mesopolyploid Brassiceae are evolutionarily distinct but functionally compatible

Yue Hao[1], Makenzie E. Mabry[2], Patrick P. Edger[3,4], Michael Freeling[5], Chunfang Zheng[6], Lingling Jin[7], Robert VanBuren[3,8], Marivi Colle[3], Hong An[2], R. Shawn Abrahams[2], Jacob D. Washburn[9], Xinshuai Qi[10], Kerrie Barry[11], Christopher Daum[11], Shengqiang Shu[11], Jeremy Schmutz[11,12], David Sankoff[6], Michael S. Barker[10], Eric Lyons[13,14], J. Chris Pires[2,15] and Gavin C. Conant[1,16,17,18]

Supplemental Table S1. Model optimization and likelihoods.

Supplemental Fig S1. Final ln likelihoods of three different topologies of the four species *B. rapa*, *B. oleracea*, *S. alba* and *C. hispanica*.

Supplemental Fig S2. Shared synteny blocks across four genomes.

Supplemental Fig S3. Species-specific and shared posterior probabilities of all 14,050 loci.

Supplemental Fig S4. Selective constraints of retained triplets partitioned into subgenomes.

Supplemental Fig S5. PANTHER Biological Processes and Molecular Functions for the Arabidopsis orthologs of genes that returned to single copy at the root branch with FDR ≥ 0.05.

Supplemental Fig S6. Number of edges connecting nodes with single copy genes that are from the same subgenome in both protein-protein interaction network and metabolic network.

Supplemental Fig S7. *Brassica rapa* subgenome assignment and genes under selective sweep.

Supplemental Table S1. Model optimization and likelihoods.

Test for	Order	Model	Topology	Total breaks in dataset	Final ln likelihood
Orders	FourSpp_M0Opt4	WGT_3rate_G1Dom_model	BrBoSaCh_Top3	5236	-68852.05
	FourSpp_M1Opt1	WGT_3rate_G1Dom_model	BrBoSaCh_Top3	5236	-68852.05
	FourSpp_M2Opt3	WGT_3rate_G1Dom_model	BrBoSaCh_Top3	5237	-68856.80
	FourSpp_M0Opt3	WGT_3rate_G1Dom_model	BrBoSaCh_Top3	5255	-68870.21
Ancestral orders	FourSpp_ANCTEST	WGT_3rate_G1Dom_model	BrBoSaCh_Top3	16854	-44505.97
	FourSpp_AncM1Opt2	WGT_3rate_G1Dom_model	BrBoSaCh_Top3	13627	-43049.68
	FourSpp_AncM0Opt2	WGT_3rate_G1Dom_model	BrBoSaCh_Top3	13870	-43163.99
Topologies	FourSpp_M2Opt3	WGT_3rate_G1Dom_model	BrBoSaCh_Top2	5237	-69855.10
	FourSpp_M2Opt3	WGT_3rate_G1Dom_model	BrBoSaCh_Top3	5237	**-68855.82**
	FourSpp_M2Opt3	WGT_3rate_G1Dom_model	BrBoSaCh_Top1	5237	-69653.03
Models	FourSpp_M2Opt3	WGT_Triple_Loss_model (Null_model)	BrBoSaCh_Top2	5237	-71007.55
	FourSpp_M2Opt3	WGT_Triple_Loss_model (Null_model)	BrBoSaCh_Top3	5237	**-69906.36**
	FourSpp_M2Opt3	WGT_Triple_W_DominantGenome	BrBoSaCh_Top3	5237	-69074.34
	FourSpp_M2Opt3	WGT_2rate_G1Dom_model	BrBoSaCh_Top3	5237	**-68940.78**
Root models	FourSpp_M2Opt3	WGT_3rate_G1Dom_brnspec_model	BrBoSaCh_Top3_RootSpec	5237	**-68767.74**
	FourSpp_M2Opt3	WGT_3rate_G1Dom_model r: WGT_RootModel_LF	BrBoSaCh_Top3	5237	**-68823.07**
	FourSpp_M2Opt3	WGT_3rate_G1Dom_model r: WGT_RootModel_MF1	BrBoSaCh_Top3	5237	-68843.19
	FourSpp_M2Opt3	WGT_3rate_G1Dom_model r: WGT_RootModel_MF2	BrBoSaCh_Top3	5237	-68847.01

Supplemental Figures

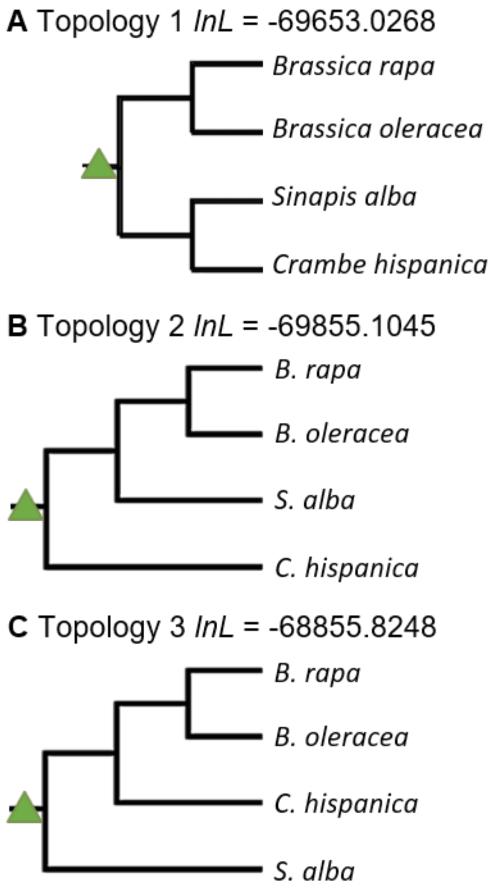

Supplemental Fig S1. Final ln likelihoods of three different topologies of the four species *B. rapa*, *B. oleracea*, *S. alba* and *C. hispanica*. The triangle indicates shared hexaploidy ancestry.

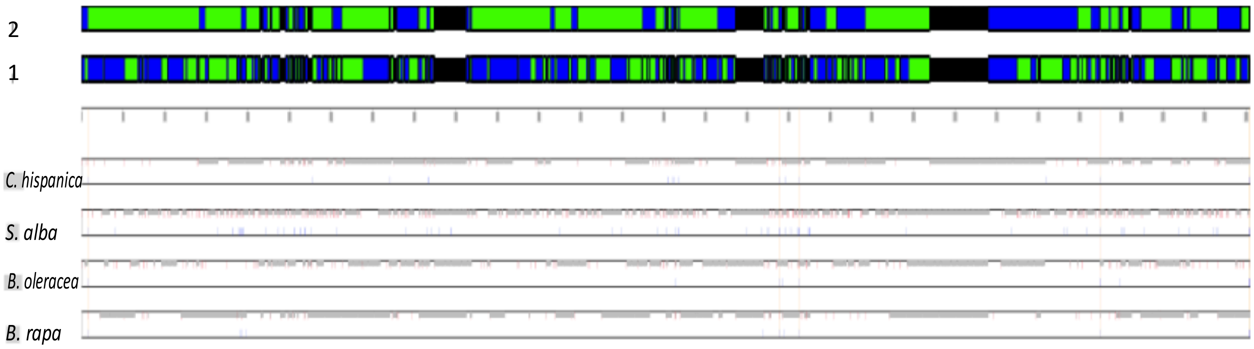

Supplemental Fig S2. Shared synteny blocks across four genomes. The green and blue blocks indicate shared parental subgenome assignment between at least three (lower blocks) or two (upper blocks) genomes with confidence > 0.85. Each change of color indicates a new block of genes with consistent assignments to the three subgenomes. Black areas indicate a lack of agreement in parental subgenome assignments. The four separate panels below show the POInT subgenome assignment in each species. Red ticks indicate switch in subgenome assignment, grey ticks indicate parental subgenome assignment confidence < 0.85 and blue ticks indicate full synteny breaks in that genome relative to the inferred ancestral order.

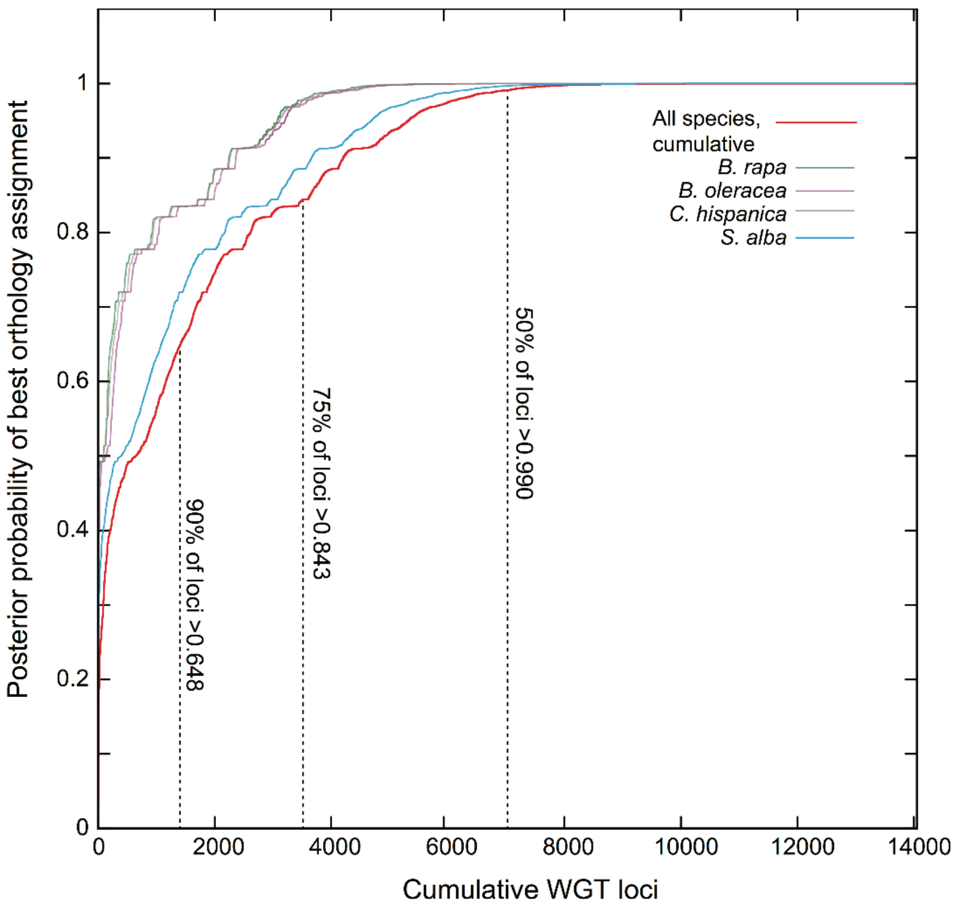

Supplemental Fig S3. Species-specific and shared posterior probabilities of all 14,050 loci. 50% of the loci have posterior probabilities larger than 0.99, 75% of the loci have posterior probabilities larger than 0.843, 90% of the loci have posterior probabilities larger than 0.648.

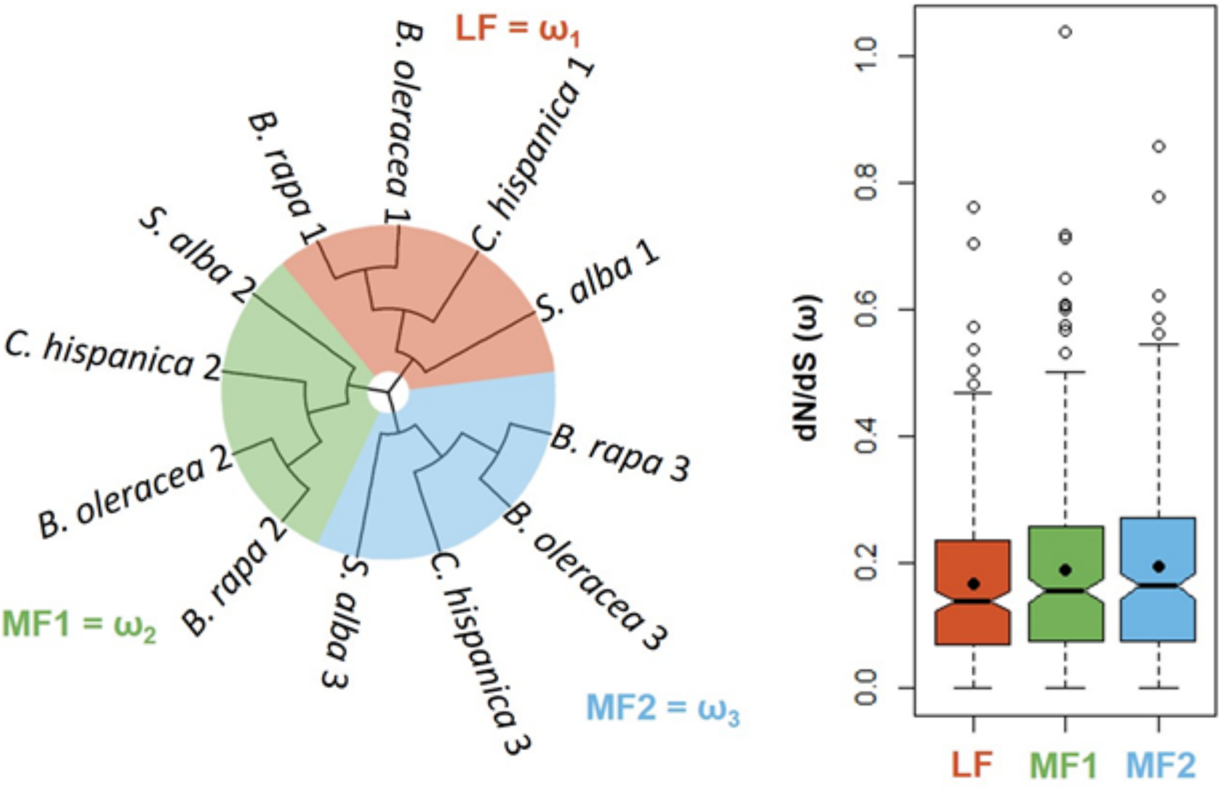

Supplemental Fig S4. Selective constraints of retained triplets partitioned into subgenomes. As shown in the schematic gene tree, three separate d_N/d_S values were estimated using codeml for each subtree containing four gene copies that were assigned to the same subgenome in four species. Notched box plots show the distributions of d_N/d_S for retained copies in each subgenome, LF, MF1 and MF2. The notches show the medians and the 95% confidence intervals. The black dots show the mean values. Pairwise Wilcoxon Rank Sum Tests (Mann and Whitney, 1947) were performed to compare the median selective constraints for retained triplets across subgenomes.

LF – MF1: $P = 0.300$

LF – MF2: $P = 0.079$

MF1 – MF2: $P = 0.516$

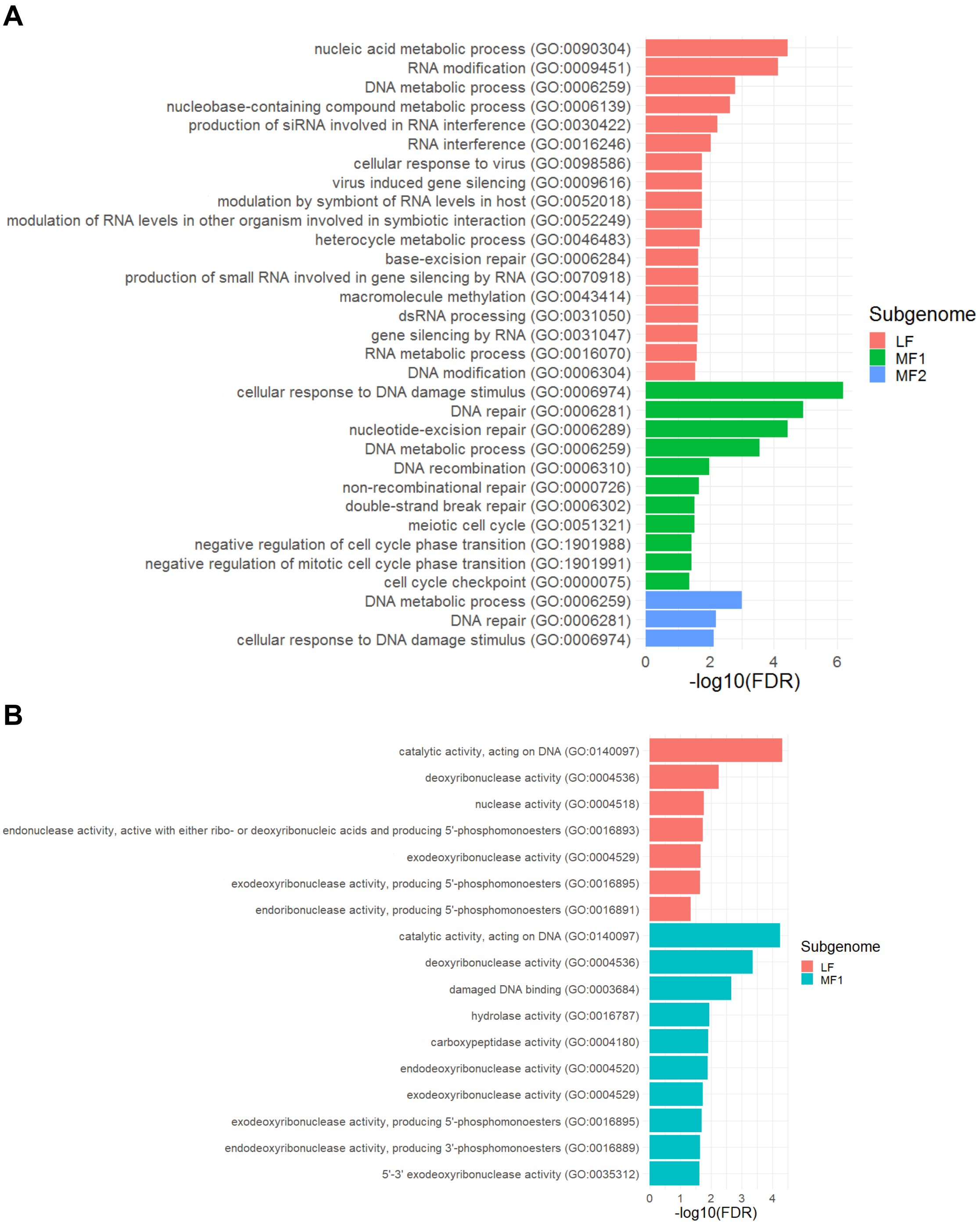

Supplemental Fig S5. PANTHER Biological Processes (*A*) and Molecular Functions (*B*) for the *Arabidopsis* orthologs of genes that returned to single copy at the root branch with FDR ≥ 0.05. The target lists are single copy genes from three subgenomes LF, MF1 and MF2. The background list was set to be all the retained duplicates and triplets.

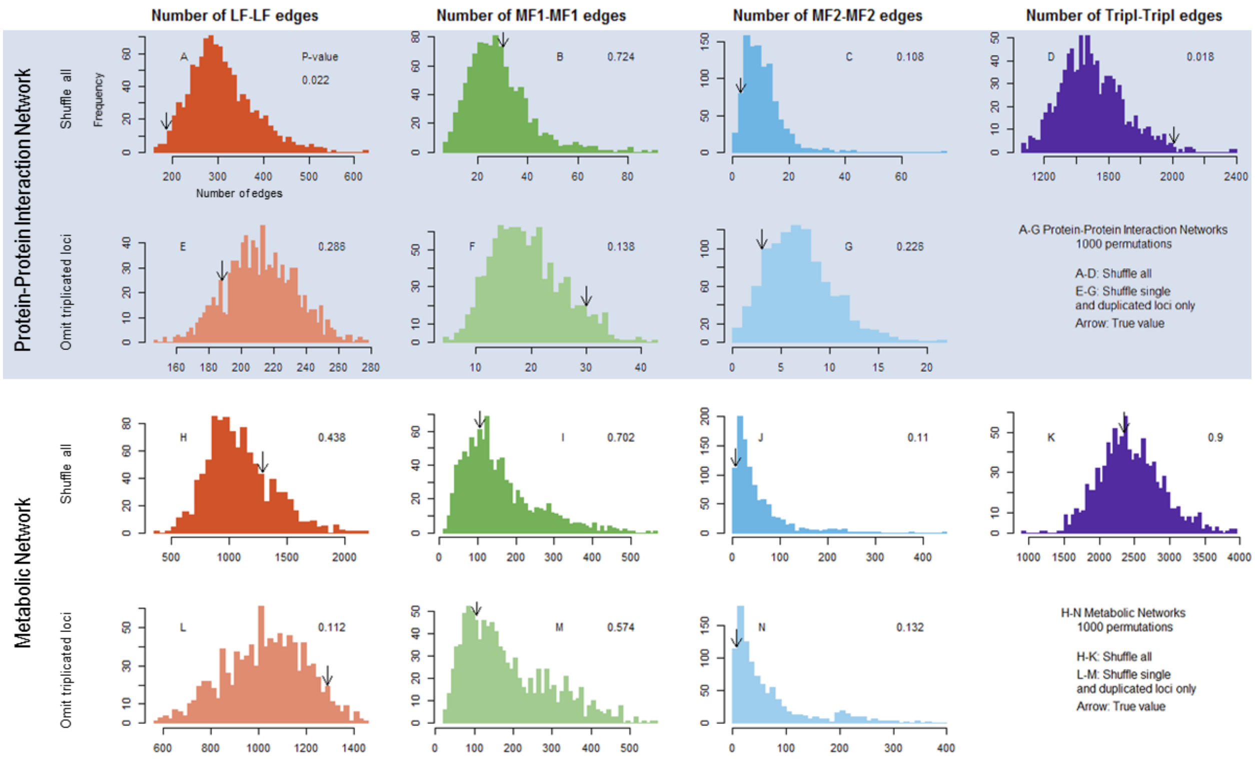

Supplemental Fig S6. Number of edges connecting nodes with single copy genes that are from the same subgenome in both protein-protein interaction network and metabolic network.

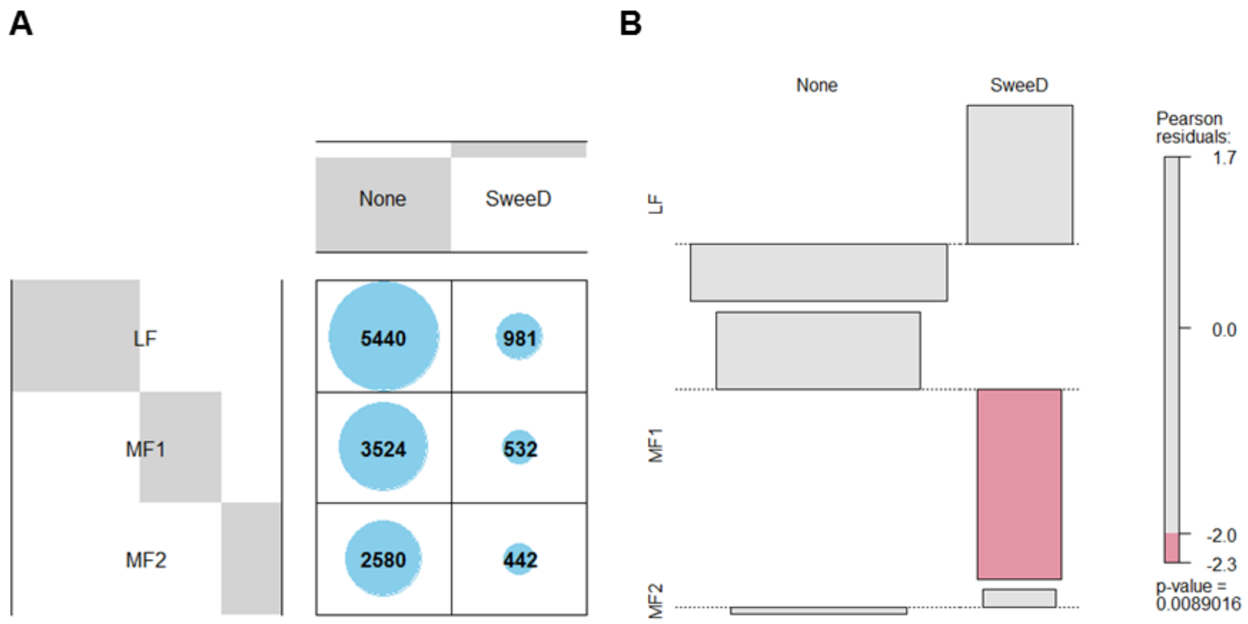

Supplemental Fig S7. *Brassica rapa* subgenome assignment and genes under selective sweep. (*A*) The number of genes from the three subgenomes (with 0.95 subgenome assignment confidence) versus selective sweeps. (*B*) The association plot based on the contingency table in *A*. The red color in the association plot indicates that the observed value is lower than expected under the random assumption. P-value (0.0089) is from chi-squared test.
